# Quality of virtual-non-contrast phases derived from arterial and delayed phases of fast-kVp switching dual-energy CT in patients after endovascular aortic repair

**DOI:** 10.1007/s10554-023-02887-x

**Published:** 2023-06-14

**Authors:** Wojciech Kazimierczak, Natalia Kazimierczak, Zbigniew Serafin

**Affiliations:** 1 Collegium Medicum, Nicolaus Copernicus University in Torun, Jagiellońska 13-15, 85-067 Bydgoszcz, Poland; 2 Kazimierczak Ortodoncja i Radiologia, Dworcowa 13/u6a, 85-009 Bydgoszcz, Poland

**Keywords:** Computed tomography angiography, Endoleak, Stents, Abdominal aortic aneurysm, Algorithms

## Abstract

Objective of this study is: to analyze CT numbers in arteries and endoleaks in true non-contrast (TNC) and virtual non-contrast phases derived from arterial (VNCa) and delayed (VNCd) phases of dual-energy CT (DECT) in patients after endovascular aneurysm repair (EVAR); to assess the impact of image noise on subjective image quality parameters and the degree of subtraction of calcifications; to calculate effective dose (ED) reduction following replacement of TNC with VNC. The study included 97 patients after EVAR procedure. An initial single-energy TNC acquisition was followed by two DECT acquisitions. CT numbers of TNC, VNCa, VNCd were analyzed statistically. VNCd images were assessed qualitatively. The mean densities in endoleaks were 46.19 HU in TNC, 51.24 HU in VNCa, 42.24 HU in VNCd. The differences between them were statistically significant (p < 0.05). The mean signal-to-noise ratio (SNR) measured in the aorta and endoleaks was highest in VNCa, lowest in TNC images. No correlation between image noise, the results of qualitative analysis of VNCd, and the degree of subtraction of calcifications was found. Omitting TNC led to mean 6.54 ± 1.63 (SD) mSv (23.28% of total examination) ED reduction. VNC images have a higher SNR compared to TNC images with significant differences in the CT numbers between the TNC and VNC reconstructions. Image noise has no impact on the subjective image quality and the degree of subtraction of calcifications in VNCd images. The findings show a high diagnostic value of VNC images and suggest that VNCd images are optimal in the assessment of endoleaks with possible substantial ED reduction.

## Introduction

Abdominal aortic aneurysms (AAAs) are a common health problem that affects up to approximately 7.6% of the male population [1]. Reduction of postoperative mortality has led to the adoption of endovascular aneurysm repair (EVAR) as the preferred method of treating AAAs [2]; however, EVAR patients need lifelong surveillance to diagnose possible life threating complications, such as stent-graft migration and endoleak [2, 3]**.**

Computed tomography angiography (CTA) and color Doppler ultrasound are currently leading methods in the surveillance of patients with acute and chronic aortic disease, as well as those after EVAR procedure. One of the most promising methods of CTA data acquisition is dual-energy CTA (DECTA). DECTA allows acquisition of two simultaneous or nearly simultaneous datasets (depending on the scanning technique) acquired in different energies. Differences between attenuation levels in the acquired datasets enable differentiating materials and elements (e.g. iodine, calcium) in the scanned area and creating maps of their occurrence [4]. In the subsequent step, it enables subtraction of iodine and reconstruction of the so-called virtual non-contrast (VNC) phase. This may allow omission of the true non-contrast (TNC) acquisition and a substantial reduction in the radiation dose [5–12]. Studies cited above confirmed the possibility of obtaining images similar in quality to TNC and the validity of using VNC in patients after EVAR. The use of iodine subtraction algorithms was, however, associated with differences between TNC and VNC images. In these studies, VNC was obtained only from the delayed phase of the study. It is, therefore, reasonable to compare the images of TNC and VNC obtained from the arterial phase (VNCa) and the delayed (VNCd) study. To our knowledge, only one study analyzing differences between TNC, VNCa, and VNCd in patients after EVAR has been conducted, but it was performed using a specific data acquisition method (dual-source scanner, Siemens Healthineers) [13].

The aim of the study was to compare TNC, VNCa, and VNCd obtained with the fast-kVp-Switching technique (GE Healthcare) by assessing the effect of image noise on the subjective image quality parameters and by performing measurements of CT numbers with the assessment of the signal-to-noise ratio (SNR). Moreover, the possible reduction of the radiation dose with the use of VNC was calculated.

## Materials and methods

### Patient population

The ethics committee of our University approved this prospective study and all patients provided written informed consent. The study included 97 CT scans of 97 patients (82 men, 15 women, mean age 72.3 years) after EVAR procedure obtained between August 2019 and December 2020. Sixty-nine patients underwent classic endovascular stent-graft implantation into the AAA, 28 patients underwent branched or fenestrated stent-graft implantation (br/fEVAR). A follow-up examination was conducted in every patient 1 month after stent graft implantation. Prior to VNC evaluation, totally 57 endoleaks were diagnosed in low level 40 keV Virtual Monoenergetic Images (VMI) evaluation. Endoleaks diagnosed in the study are summarized in Table 1. The exclusion criteria were: known adverse reactions to iodinated contrast media and impaired renal function (glomerular filtration rate < 30 mL/min).

**Table 1:** Summary of endoleaks diagnosed

Endoleak type	Biphasic (VNCd + VMI arterial, delayed)	
	n	%
Ia	4	7.02
Ib	2	3.51
II	39	57.14
III	12	21.43
Total	57	

*VNCd* virtual non-contrast derived from delayed phase, *VMI*  virtual monoenergetic images

### Scanning parameters, dose evaluation

A standard triphasic examination protocol was used with the delay of 60 s between the arterial and delayed phases. All CT scans were obtained using a dual-energy fast kVp switching scanner (Discovery 750 HD, GE Healthcare). Scanning parameters in both TNC and VNC data acquisitions were as follows: a detector configuration of 128 × 0.6 mm, a pitch of 0.984, gantry rotation time of 0.6 s.

TNC phase was obtained using single energy, voltage was 120 kVp, an automatic dose adjustment system was used, Noise Index was set to 30.0, mAs range 100–200 mAs. Both VNCa/d phases were obtained using the fast-kVp switching technique. The GSI-40 preset was used, intended for abdominal examinations with the default radiation exposure level of 12.09 mGy per tube rotation, tube current 360 mAs.

Intravenous administration of 80 mL of iohexol (Ominpaque 350, GE Healthcare) to the peripheral vein at a rate of 4 mL/min was performed. non-ionic iodine contrast agent was used. The bolus tracking tool was used, triggering the start of arterial acquisition once 125 HU was exceeded in the region of interest (ROI) positioned in the descending aorta (examinations covering the thorax) or at the level of the superior mesenteric artery (examinations limited to the abdominal cavity and pelvis). The delayed phase was performed automatically 60 s after the onset of the arterial phase. The scan coverage was from clavicles to greater trochanters or from diaphragm to greater trochanters depending on vascular surgeon referral.

Effective dose (ED) is the product of the dose-length product of each phase and the conversion coefficient (k). The assumed k coefficient was 0.015 mSv/mGy × cm [14]. The possible ED reduction was analyzed by computing the ED of triphasic and biphasic (both post-contrast acquisitions + VNC) study protocols.

### Quantitative image analysis

All images were viewed on a dedicated GE Healthcare console (GSI Viewer, Advantage Workstation Release 4.6, GE Healthcare), enabling the analysis of dual-energy studies. Comparisons of the attenuation and noise of TNC, VNCa, and VNCd images were made by drawing circular ROIs in the aorta in the main stent-graft module, aneurysm thrombus, one of the common iliac arteries, subcutaneous adipose tissue, psoas muscle, and endoleaks (if present). An automatic ROI propagation tool was used, and ROIs were made as large as possible, avoiding calcifications, plaques, and artifacts. Mean attenuation and image noise (defined as SD) were registered. SNR was calculated from the formula:

$${\textbf{SNR}} = {\text{A}_{\text{A,E}}}/{\text{N}}$$

where A_A_ is the mean attenuation of the artery lumen (aorta, common iliac artery), A_E_ is the mean attenuation of the endoleak lumen, N is noise (SD in the subcutaneous adipose tissue).

### Qualitative image analysis

The differences between TNCs and VNCs, which were apparent at first glance, ruled out the possibility of blinding the reader to the type of displayed images. Subjective image quality of TNC and VNC was assessed with criteria:

1. Diagnostic value of the images on a 5-point scale: 1—undiagnostic images, 2—images of low diagnostic quality, 3—images of acceptable diagnostic quality, 4—images of good diagnostic quality, 5—images of excellent diagnostic quality.
2. Calcifications within the aneurysm on a 4-point scale: 1—no calcifications, 2—single, point, peripheral calcifications, 3—circular calcifications or calcifications within thrombus, 4—massive circular calcifications and calcifications within thrombus.
3. The degree of calcification subtraction on a 5-point scale: 1—no subtraction, 2—minimal degree of calcification subtraction, 3—intermediate degree of minor calcification subtraction, 4—significant degree of large calcification subtraction, 5—total subtraction of calcifications.
4. The final subjective assessment of the applicability of VNC on a 3-point scale: 1—image fully acceptable, 2—image acceptable with reservations, 3—image unacceptable.

TNC and VNCd were subjectively analyzed and compared. Impact of the image noise on the above-mentioned criteria was analyzed.

### Statistical analysis

The analysis of quantitative variables (i.e. expressed in number) was performed by calculating the mean, standard deviation, median and 1.3 quartiles. The analysis of qualitative (i.e. non-numeric) variables was performed by calculating the number and percentage of occurrences of each value. Comparisons of the values of quantitative variables were performed using the Mann–Whitney or Kruskal–Wallis tests. After detecting statistically significant differences, post-hoc analysis with Dunn’s test was performed to identify statistically significant differences between groups. Comparison of the quantitative variables in the two repeated measurements was performed using the Wilcoxon test. Comparison of the quantitative variables in three and more repeated measurements was performed using the Friedman test. After detecting statistically significant differences, a post-hoc analysis (Wilcoxon’s paired tests with Bonferroni correction) was performed to identify statistically significant differences between measurements. Comparison of the qualitative variables in two repeated measurements was performed using the McNemar test. A significance level of 0.05 was adopted in the analysis. All p values below 0.05 were interpreted as indicative of significant correlations. The analysis was performed using Google Sheets, Microsoft Excel and R software, version 4.1.1.

## Results

### Quantitative analysis

**Fig. 1:**
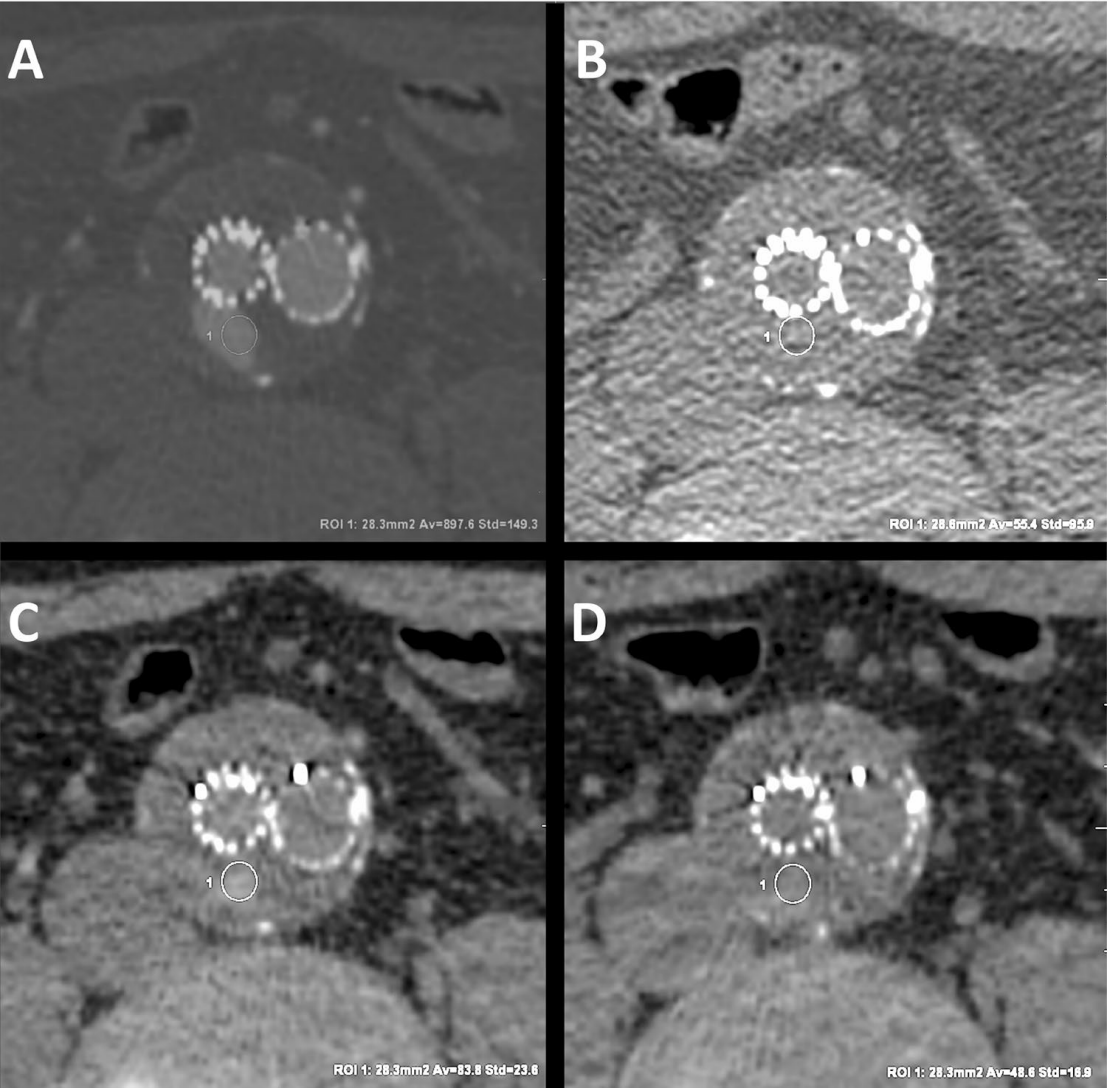
Images presenting differences in CT numbers (average ± SD) in the region of type II endoleak in arterial, true non-contrast (TNC) and 2 virtual non-contrast (VNC) phases. Reconstructions: 40 keV VMI arterial phase (**A**—897 ± 149 HU), TNC (**B**—55 ± 95 HU), VNCa (**C**—83 ± 23 HU), VNCd (**D**—48 ± 16 HU). Same axial slice, same patient. TNC, VNCa, VNCd images presented with the same window settings (W 350, L 40)

Quantitative analysis of TNC and both VNC phases revealed significant differences in the densities of arteries and endoleaks. The differences in the mean results of density measurements were as high as 13.08 HU for the aorta, 8.55 HU for the iliac artery, and 11.42 HU for endoleaks. In all indicated cases, the mean arterial or endoleak density was significantly higher in VNCa than in VNCd. In the case of other tissues, iodine subtraction during the reconstruction of the VNC phases did not significantly affect the degree of radiation absorption—the difference between the mean density was 0.27 HU in the thrombus, 2.24 HU in the psoas muscle, and 0.72 HU in fat tissue. Measurements showed repeatable occurrence of higher CT attenuation numbers in VNCa compared to TNC within the structures subjected to intense contrast enhancement (aorta, iliac artery, endoleak). In contrast, the mean results of VNCd measurements showed a lower mean density of the above structures compared to TNC. The mean densities in the aorta were: TNC 49.82 HU, VNCa 55.32 HU, VNCd 42.24 HU; in endoleaks: 46.19 HU, 51.24 HU, 39.82 HU, respectively. These differences were statistically significant (p < 0.05). An example of the high differences in the CT numbers measured in endoleak is shown in Fig. 1.

The mean noise level of the examined structures was significantly higher in TNC images, and in the case of measurements in the aorta, thrombus, psoas muscle, fat tissue, iliac arteries, and endoleaks, the mean noise level was in the range of 51.29–62.21. Measurements made within the above-mentioned structures in VNC reconstructions showed a significantly lower average noise level, ranging from 23.15 to 27.35 in VNCa and from 22.97 to 37.28 in VNCd. A summary of measurements performed is presented in Table 2. The differences in the mean densities of structures are summarized in Table 3.

**Table 2:** Mean attenuation, noise ± SD, analysis of statistical significance of differences in the densities of chosen structures

Structure	Phase	Mean attenuation ± SD [HU]	Mean noise ± SD [HU]	p
Aorta	TNC	49.82 ± 13.19	62.21 ± 19.42	p < 0.001
	VNCa	55.32 ± 18.21	24.67 ± 9.44	
	VNCd	42.24 ± 11.14	25.36 ± 8.72	VNCa, TNC > VNCd
Thrombus	TNC	36.81 ± 10.09	54.71 ± 13.21	p < 0.001
	VNCa	24.7 ± 11.93	26.52 ± 6.98	
	VNCd	24.43 ± 9.72	27.22 ± 6.94	TNC > VNCa, VNCd
Psoas muscle	TNC	46.94 ± 10.12	60.12 ± 9.99	p < 0.001
	VNCa	34.64 ± 8.96	26.11 ± 6.57	
	VNCd	36.88 ± 10.15	27.18 ± 6.81	TNC > VNCd > VNCa
Adipose tissue	TNC	− 101.18 ± 21.25	51.29 ± 26.82	p = 0.001
	VNCa	− 104.56 ± 11.8	27.35 ± 6.37	
	VNCd	− 105.28 ± 12.48	27.28 ± 7.25	TNC > VNCd
Common iliac artery	TNC	46.24 ± 13.25	54.29 ± 14.98	p < 0.001
	VNCa	54.14 ± 17.35	24.47 ± 7.63	
	VNCd	45.59 ± 12.8	25.57 ± 9.04	VNCa > TNC, VNCd
Endoleaks	TNC	46.19 ± 20.76	61.06 ± 40.86	p = 0.002
	VNCa	51.24 ± 27.97	23.15 ± 11.42	
	VNCd	39.82 ± 18.29	21.96 ± 10.10	VNCa > VNCd

*TNC* true non-contrast phase, *VNCa* virtual non-contrast arterial, *VNCd* virtual non-contrast delayed

**Table 3:** Differences in mean densities between structures in TNC and VNCa/d reconstructions

Structure	Δ VNCa – VNCd [HU]	Δ TNC – VNCa [HU]	Δ TNC – VNCd [HU]
Aorta	13.08	− 5.5	7.58
Thrombus	0.27	12.11	12.38
Psoas muscle	− 2.24	12.3	10.06
Adipose tissue	0.72	3.38	4.1
Common iliac artery	8.55	− 7.9	0.65
Endoleaks	11.42	− 5.05	6.37

Data are mean values

*TNC* true non-contrast phase, *VNCa* virtual non-contrast arterial, *VNCd* virtual non-contrast delayed

The mean SNR in VNCa measured in the aorta, iliac artery, and endoleaks were 2.11, 2.08, and 2.04, respectively, and were significantly higher than that in TNC (1.09, 1.02, 1.08, respectively). SNR measured in VNCd showed intermediate values between TNC and VNCa (1.5, 1.64, and 1.44 in aorta, iliac artery, endoleaks, respectively). The results of the SNR level calculations are summarized in Table 4.

**Table 4:** Mean SNR in arteries and endoleaks

Structure	Phase	SNR ± SD
Aorta	TNC	1.09 ± 0.45
	VNCa	2.11 ± 0.71
	VNCd	1.54 ± 0.62
Common iliac artery	TNC	1.02 ± 0.51
	VNCa	2.08 ± 0.77
	VNCd	1.67 ± 0.74
Endoleaks	TNC	1.08 ± 0.61
	VNCa	2.04 ± 1.25
	VNCd	1.45 ± 0.76

Data are mean values ± SD

*TNC* true non-contrast phase, *VNCa* virtual non-contrast arterial, *VNCd* virtual non-contrast delayed, *SNR* signal-to-noise ratio

### Qualitative analysis

**Fig. 2:**
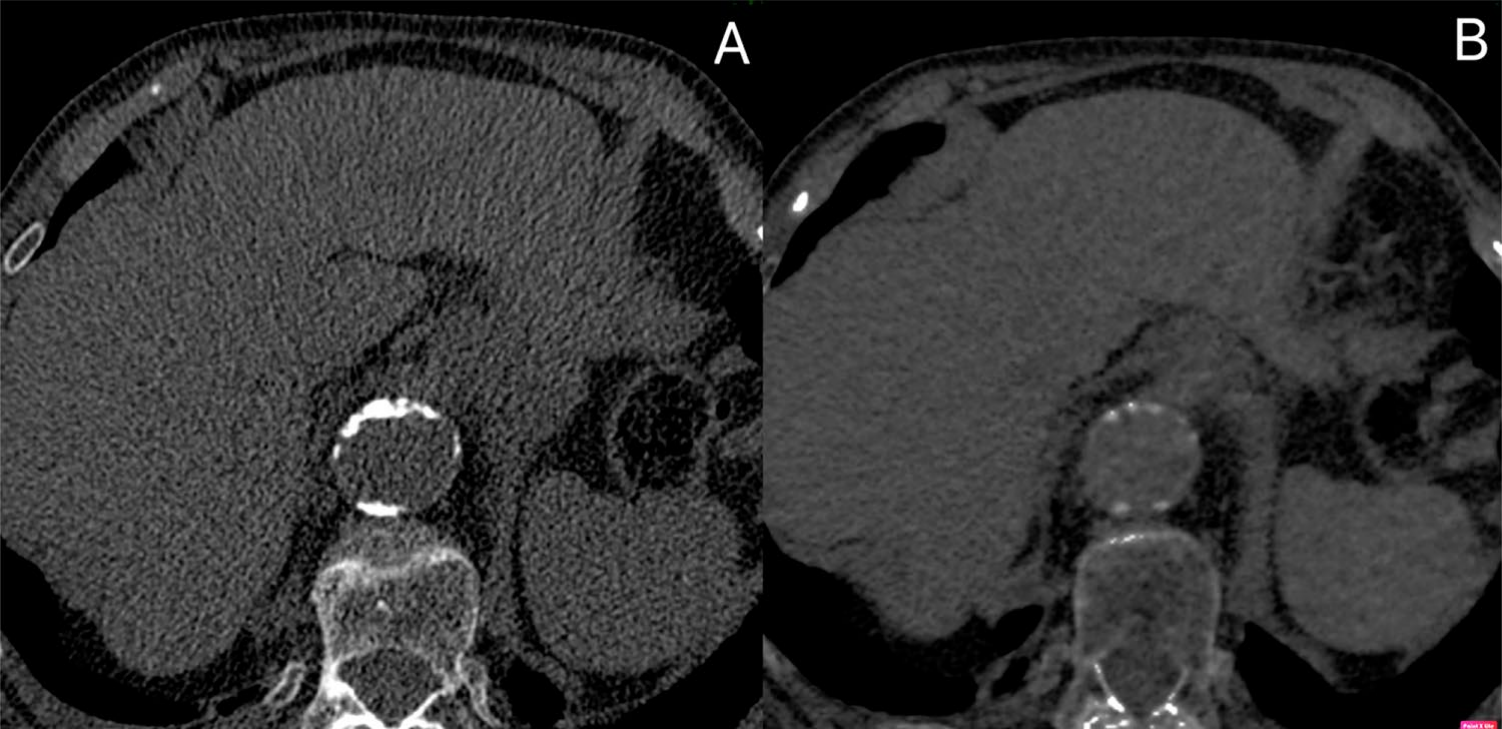
Significant calcification subtraction in VNCd reconstruction (the same level of aorta, **A—**TNC images, **B**—VNCd)

All 97 TNC datasets contained calcifications within the aneurysm sac. All VNCd datasets revealed erroneous subtraction of calcifications, with 45 (46.39%) cases marked as intermediate and 4 (4.12%) as significant. An example of a significant level of subtraction of calcifications is given in Fig. 2.

Qualitative assessment of the examinations marked all VNCd datasets as acceptable in post-EVAR evaluation—fully in 41 (42.27%) cases and with restrictions in 56 (57.73%) cases.

The results of the qualitative assessment of the VNCd and TNC images are summarized in Table 5.

**Table 5:** Results of the qualitive assessment of VNCd and TNC images

Parameter	No. (%)
TNC—image quality	
Undiagnostic images	0 (0.00%)
Low	0 (0.00%)
Acceptable	0 (0.00%)
Good	74 (76.29%)
Excellent	23 (23.71%)
TNC—calcifications	
None	0 (0.00%)
Single, punctate, peripheral	45 (46.39%)
Circumferential or within thrombus	46 (47.42%)
Circumferential, massive and within thrombus	6 (6.19%)
VNC—image quality	
Undiagnostic images	0 (0.00%)
Low	2 (2.06%)
Acceptable	30 (30.93%)
Good	65 (67.01%)
Excellent	0 (0.00%)
VNC—level of calcification subtraction	
None	0 (0.00%)
Minimal	48 (49.48%)
Intermediate of minor calcifications	45 (46.39%)
Significant of large calcifications	4 (4.12%)
Total	0 (0.00%)
VNC—final assessment	
Fully acceptable	41 (42.27%)
Acceptable with reservations	56 (57.73%)
Unacceptable	0 (0.00%)

Data in brackets are percentages of total number of examinations (97)

*TNC* true non-contrast phase, *VNC* virtual non-contrast

The impact of image noise on the qualitative image parameters was evaluated. The analyses performed showed no statistically significant correlation (p > 0.05%) between:

- noise and the subjective assessment of diagnostic quality;
- noise and the degree of subtraction of calcifications;
- noise and the subjective assessment of the possibility of using VNCd.

A summary of the above-mentioned correlations is presented in Table 6.

**Table 6:** Correlations between image noise (SD) and image quality, level of calcification subtraction and final assessment of the possibility of using VNCd

Parameter	Group	VNCd SD [HU]—Aorta			p
		Mean ± SD	Median	Quartiles	
VNC—image quality	Low or acceptable (N = 32)	25.92 ± 8.15	24.25	20.78–30.7	p = 0.137
	Good (N = 65)	24.05 ± 10.01	22.2	18.5–25.4	
VNC—level of calcifications subtraction	Minimal (N = 48)	25.46 ± 11.25	22.35	19.38–25.27	p = 0.991
	Intermediate (N = 45)	23.95 ± 7.43	22.5	17.1–28.2	
	Significant (N = 4)	23.18 ± 6.05	23.1	20.62–25.65	
VNC—final assessment	Fully acceptable (N = 41)	23.39 ± 7.66	22.3	19.3–24.1	p = 0.504
	Acceptable with reservations (N = 56)	25.6 ± 10.52	22.65	18–30.45	

*TNC* true non-contrast phase, *VNC* virtual non-contrast

### Radiation dose

The ED of the full triphasic protocol was 27.95 ± 5.06 (SD) mSv (range 18.35–38.00 mSv). The TNC dose was 6.54 ± 1.63 (SD) mSv. Omitting the TNC phase with the use of VNCd reconstructions could lead to 23.28% reduction of examination ED.

## Discussion

The VNC images obtained in this study were characterized by a higher SNR compared to the TNC images. There were significant differences in the densities of vascular structures and endoleaks in the TNC, VNCa, and VNCd reconstructions, with significantly lower mean densities in VNCd. The above correlations are concordant with part of the literature [13, 15].The qualitative and quantitative analyses performed showed that VNCd images may be a better approximation of TNC images than VNCa images in the assessment of endoleaks, and their use enables a reduction of the radiation dose.

The presence of hyperdense calcifications within the aneurysm sac, subtracted in VNC, may lead to a false positive recognition of endoleaks. This phenomenon has already been described in the literature, and the degree of subtraction was noted as absent/mild [5, 6, 8] or was not assessed [9–11]. In our analysis, the subjective degree of subtraction of calcifications was determined in most cases as minimal or intermediate, and in 4% of cases, the level of calcification subtraction was defined as significant. Statistical analyses did not show a significant correlation between image noise and the degree of subtraction of calcifications. Moreover, image noise did not significantly affect the subjective assessment of the quality of the images, as well as the level of acceptance of the images in the assessment of the presence of endoleak.

The analysis of the ROI measurements showed significant differences in the CT numbers of the structures undergoing intense contrast enhancement, depending on the phase of the study, from which VNC images were reconstructed. The VNCa images showed a significantly higher densities of ROI located within the aorta, iliac artery, and endoleak compared to the TNC and VNCd images. The phenomenon of statistically significant differences in the degree of radiation absorption between TNC and VNC within arteries has already been described in the literature [13, 15, 16]. There are also publications in which no statistically significant differences in the attenuation of abdominal aorta and other abdominal vessels was found between TNC and VNC images [6, 8]. The issue of differences in tissue imaging between TNC and VNC images acquired in DECT systems has been examined by several researchers, including Sauter et al. [17]. In their study, they found no significant differences between tissues, except for spongious bone, when comparing TNC and VNC reconstructions [17]. However, they did find differences above 15 HU in 19% of aortic measurements. Similar results were found in previously mentioned studies [13, 15, 16]. The authors of the study concluded that iodine subtraction algorithms depend on DECT data acquisition, and that dual layer DECT systems are more capable of precise iodine subtraction than other dual-energy systems.

The previously mentioned study by Lehti et al. [13] showed a tendency similar to the one obtained in our research—the occurrence of a higher degree of radiation absorption within arteries in VNCa images, with a similar degree of radiation absorption in muscles and adipose tissue. The phantom study performed by Toepker et al. [15] also showed differences in vessel densities in VNCa and VNCd, with higher mean values of radiation absorption in VNCa than in VNCd and TNC. Therefore, it can be assumed that the algorithms processing VNC images, in some cases, are unable to properly quantify iodine present in high concentrations in the examined structures. The phenomenon of a higher mean density of areas identified later as an endoleak in VNCa than in TNC may potentially be important in the process of identifying endoleaks, thus making their diagnosis difficult and reducing the diagnostic value of the examination if biphasic examination is used with VNCa reconstruction. On the other hand, since endoleaks in VNCd have lower densities than in TNC, this could potentially result in a better enhancement of the presence of endoleaks during the comparison of VNCd images and the delayed phase of the study. Further research should focus on the influence of the type of the dual-energy acquisition method and software version used on this phenomenon. A separate issue is the possibility of using VNC reconstructions in a variety of clinical applications, in which it is essential to assess differences in structure densities in the subsequent stages of the study (e.g. assessment of adrenal adenomas, liver and kidney lesions).

The DECT system used in our study had a major limitation, which was the need for prospective determination of the examination protocol, along with the risk of using a higher radiation dose. Dual-source and sequential DECT systems default to single-energy mode, which precludes acquiring spectral data and thus VNC reconstructions [18]. Fortunately, split-filter, multi-layer detectors DECT systems, as well as photon-counting detectors (PCD), are free from such limitations, allowing for retrospectively acquiring DE datasets and spectral reconstructions. In our opinion, such systems allow for broader and more frequent use of reconstructions such as VMI and material decomposition algorithms (VNC, virtual non-calcium). The use of these types of reconstructions significantly increases the diagnostic value of angiographic examinations and brings significant benefits to the patient [18–20]. Moreover, frequent use of these tools allows for their expert use and application in particularly useful clinical applications.

The recently PCD CT systems, after the introduction of multi-row and DECT systems, are a significant advancement in medical CT scanners. PCD CT systems are able to preserve and quantify the energy of each photon and generate spectral reconstructions of wide range [21]. The 2022 publication by Decker et al. demonstrated the high diagnostic value of VNC phase reconstructed with PCD (VNCpc) in post-EVAR CT assessment [22]. VNCpc images derived from CT angiographies of the aorta showed potential as a substitute for TNC images in follow-up scans after EVAR. Expert readers rated 95% of VNCpc images as suitable for replacing TNC-series. VNCpc images demonstrated high image quality with complete aortic contrast removal and minimal erroneous subtraction of stent parts or calcifications. They also showed lower image noise, higher SNR, and smaller CT-value differences to TNC-series than conventional DECT-acquired VNC series. These findings are accompanied by results of another study performed in 2022 by Mergen et al. [23]. The authors found no significant difference in errors between arterial or portal venous VNC reconstructions (3.3 HU vs 3.5 HU, P = 0.16). Subjective image quality was rated lower in VNC images, but diagnostic quality was reached in 99–100% of patients. The study concluded that abdominal virtual noncontrast images from the arterial and portal venous phase of PCD CT yielded accurate CT attenuation and good image quality compared with true noncontrast images, although combined with higher noise values. The above-mentioned studies are supported by a few other, showing comparable results [24, 25]. However, there were also papers published, showing significant differences in CT numbers in TNC and VNCpc images [26]. Similar to DECT systems, these discrepancies in research results suggest the need for further work on VNC reconstruction algorithms, which likely reveal vendor- and software-specific differences. However, these promising results, combined with the ability of PCD systems to generate spectral and VNC reconstructions of each scan, allow us to believe that the widespread use and development of DECT and PCD systems will enable routine application of VNC reconstructions on a global scale, leading to a significant reduction in radiation doses received by patients during CT scans.

### Limitations

Conclusions based on the results of the examination carried out with hardware and software from one manufacturer (GE Healthcare) with a specific type of dual energy acquisition (fast kVp switching) are both an advantage and a limitation of our study. This issue may explain, inter alia, the differences in the densities of VNCa, VNCd, and TNC compared to the data from the literature. This means that the study results are appropriate for only one dual energy acquisition technique and probably one version of the CT scanner and software.

Another limitation that should be pointed out is the dependence of ED on the scanning parameters. Those used in our protocol are suboptimal (high Noise Index), adopting lower levels of Noise Index would lead to even higher potential ED reduction.

Another potential limitation of the study was the possibility of imprecise ROI propagation within structures. It was of particular importance in the case of small structures, such as endoleaks—the different respiratory phase during the three phases of examination could cause a significant shift of the layers with the presence of a small endoleak which had an impact on the obtained values of density and SD measurements, and therefore also on the SNR coefficient. However, it should be remembered that the patient's respiratory and involuntary movements cannot be fully controlled, and 100% repeatability in the ROI placement is impossible. This problem applies to all studies on this subject.

## Conclusion

VNCa and VNCd images are characterized by a higher SNR compared to TNC images. There are significant differences in the densities of vascular structures and endoleaks in TNC, VNCa and VNCd reconstructions, with significantly lower mean densities in VNCd. No statistically significant correlation was found between image noise and the subjective image quality parameters and the degree of subtraction of calcifications. In conclusion, VNCd images are more suitable than VNCa images and sufficient as a replacement of TNC in the diagnosis of endoleaks with a possible 23.28% reduction of radiation dose.
